# Overcoming the challenges of membrane protein crystallography

**DOI:** 10.1016/j.sbi.2008.07.001

**Published:** 2008-10

**Authors:** Elisabeth P Carpenter, Konstantinos Beis, Alexander D Cameron, So Iwata

**Affiliations:** 1 Membrane Protein Laboratory, Imperial College London, Diamond Light Source Ltd., Harwell Science and Innovation Campus, Didcot OX11 ODE, United Kingdom; 2 Division of Molecular Biosciences, Membrane Protein Crystallography Group and Membrane Protein Laboratory, Imperial College, London SW7 2AZ, United Kingdom; 3 ERATO Human Receptor Crystallography Project, 3rd Floor, Building A, Graduate School of Medicine, Kyoto University, Yoshida-konoe-cho, Sakyo-ku, Kyoto 606-8501, Japan

## Abstract

Membrane protein structural biology is still a largely unconquered area, given that approximately 25% of all proteins are membrane proteins and yet less than 150 unique structures are available. Membrane proteins have proven to be difficult to study owing to their partially hydrophobic surfaces, flexibility and lack of stability. The field is now taking advantage of the high-throughput revolution in structural biology and methods are emerging for effective expression, solubilisation, purification and crystallisation of membrane proteins. These technical advances will lead to a rapid increase in the rate at which membrane protein structures are solved in the near future.

## Introduction

Membrane proteins continue to be among the most challenging targets in structural biology. All cells and organelles are contained within a hydrophobic lipid bilayer. Integral membrane proteins are embedded in the lipid bilayer, often with additional domains outside the membrane. These proteins are involved in a wide variety of biological processes including photosynthesis, respiration, signal transduction, molecular transport and catalysis.

**Figure 1:**
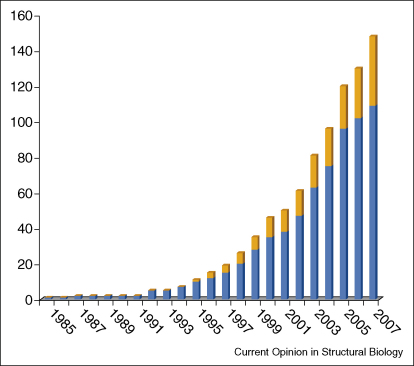
Growth of unique membrane protein structures deposited in the PDB. Proteins were found by inspection of the ‘Membrane proteins of known 3D structure’ database [4,5] (http://blanco.biomol.uci.edu/Membrane_Proteins_xtal.html), In blue, the number of structures of prokaryotic membrane proteins, and in yellow, the total number of eukaryotic structures. Both monotopic and multispanning proteins are included. For this study proteins are regarded as unique if they come from the same family but for different species. Structures are not counted in these statistics if they represent mutants, alternative conformations or ligand complexes of a previously counted structure.

Membrane proteins represent between 20 and 30% of the proteomes of most organisms [1] and more than 40% of drug targets [2] and yet very few structures of these molecules have been solved by X-ray crystallography or NMR. The first membrane protein structure was published in 1985 [3] and since then the number has increased slowly but steadily (Figure 1). To date there are over 50 000 entries in the Protein Data Bank (PDB) repository of protein structures, but less than 1% of these entries represent membrane proteins. Of the 368 membrane protein structures in the ‘Membrane proteins of known 3D structure’ database [4,5] (http://blanco.biomol.uci.edu/Membrane_Proteins_xtal.html), 148 belong to unique proteins. Eukaryotic membrane proteins are particularly underrepresented, with only 39 examples (monotopic and multispanning).

Membrane proteins are difficult to study for a number of reasons. Their surface is relatively hydrophobic and they can only be extracted from the cell membrane with detergents. They are also often flexible and unstable. This leads to challenges at all levels, including expression, solubilisation, purification, crystallisation, data collection and structure solution. This review highlights the issues associated with membrane protein structural biology and outlines recent approaches that have been successful in determining new structures.

## Expression and purification

Membrane proteins of known structure have been purified from natural sources, produced recombinantly or, in the case of short peptides, synthesised chemically. They have been successfully expressed in the bacteria *Escherichia coli* and *Lactococcus lactis*, the yeasts *Pichia pastoris* and *Saccharomyces cerevisiae*, in insect cells and in mammalian cell lines (reviewed in Junge *et al.* [6^••^]). There are a number of factors that influence the success of an expression system [7]. Production in *E. coli* is quick, relatively inexpensive and easy to use enabling many constructs to be screened quickly. Eukaryotic proteins may, however, require the use of eukaryotic systems for expression. Firstly, membrane proteins have to be targeted to the host cell membrane before they can fold correctly. Specific systems are required in the host cell such as the SRP-Sec61 system that inserts membrane proteins into the endoplasmic reticulum of eukaryotic cells [8]. Secondly, membrane proteins are embedded in lipid, and the composition of these lipids varies among the systems. The nature of the lipids can affect the stability of the protein and consequently its likelihood of crystallisation. Thirdly eukaryotic proteins may undergo post-translational modifications, such as glycosylation, and only higher eukaryotic cell lines provide the necessary machinery [6^••^]. It is often necessary to experiment with a variety of expression systems for each protein. Most of the structures of bacterial membrane proteins that are present in the PDB were successfully expressed in *E. coli*. Eukaryotic membrane proteins, however, are most commonly purified from native sources. Of the 39 unique eukaryotic membrane proteins, only 17 were produced using recombinant methods, nine of these in yeast systems (*Pichia pastoris* and *Saccharomyces cerevisiae*) [9–17], four in insect cells [18,19^••^,20] and four in *E. coli* [21–24].

Membrane proteins are extracted from the host cell membrane by the addition of detergents, which cover the hydrophobic surface of the protein, allowing solubilisation. The choice of detergent is a crucial part of the purification process. Often a series of detergents are tested and the detergent that extracts the largest quantity of soluble, active, homogeneous, stable protein is used, provided that the cost of the detergent is not limiting. However, it should be noted that some strong detergents like FOS-Choline are very efficient at extracting proteins from the membrane, but this does not guarantee stably solubilised membrane proteins. The detergent dodecyl maltoside (DDM) is often used to extract membrane proteins from the lipid bilayer as it is relatively cheap and can give stably solubilised membrane proteins [25^•^]. Protein can subsequently be exchanged into a variety of different detergents for crystallisation trials [26].

Advances are being made in developing methods that can assess the expression and purification of membrane proteins in a high-throughput manner [27,28^•^]. The use of a cleavable green fluorescent protein (GFP) with a his-tag fused to the C-terminus of the protein has proved to be very effective as a way of following the protein during purification, a technique pioneered by Prof. Jan-Willem De Gier [27] and Eric Gouaux [29^•^]. For this system to be successful in *E. coli* expression systems, the target membrane protein must have a cytoplasmic C-terminus, since the GFP can only be correctly folded and become fluorescent in the cytoplasm [30,31]. Prokaryotic and eukaryotic GFP fusion proteins expressed in *E. coli* [32^••^] and *S. cerevisiae* [33^••^,34^••^] allow rapid selection of targets with the highest expression yields for large-scale purification. In-gel fluorescence analysis and fluorescence size-exclusion chromatography (FSEC) of GFP fusion proteins clearly show whether a protein is monodisperse in particular detergents without the need for prior purification [29^•^].

An alternative method has been described to rapidly screen many constructs and conditions for expression and solubilisation of membrane proteins. The method was initially described for proteins expressed in 96-well plates, with small-scale purification in a 96-well format and detection of proteins blotted onto filters using antibodies to the purification tag [28^•^]. This method has since been modified to detect protein expression in colonies, using the colony filtration blot method [35] in which colonies are blotted onto membranes, expression is induced and the cells are lysed with test detergents. Detergent solubilised proteins are filtered through membranes and detected with antibodies against the tag.

Membrane proteins are often unstable in detergent micelles. Finding constructs or conditions where the protein is more stable can lead to improved crystallisation [36]. Sometimes, addition of lipids is essential to obtain stably solubilised samples [12,37,38^••^,39]. Screening different buffer and detergent conditions is often necessary and the aggregation state of the material can be monitored using gel filtration, electron microscopy or ultracentrifugation [40]. Another way to assess the state of a protein is to monitor the thermal stability. Stevens and co-workers have adapted a method for soluble proteins monitoring the fluorescence of a covalently bound dye attached to accessible cysteine residues [41^•^]. In a recent study Tate and co-workers [42^••^] improved the stability of the β1-adrenergic receptor by making point mutations and testing the resulting mutants for activity as a function of temperature. A number of mutations gave increased thermostability and a combination of six of these mutations gave a protein with an increase in Tm of 21 °C. This subsequently enabled the structure of this protein to be solved [43].

## Crystallisation

Protein crystallisation is a process of testing a large number of possible crystallisation reagents. Once initial crystallisation conditions are found, further optimisation is usually necessary to obtain well-diffracting crystals. The initial screen is generally completed using 96-well plates and the vapour diffusion method. In the past 10 years, the volumes required for these crystallisation experiments have been greatly reduced, so that now routine screening for crystals is achieved with 100 nl drops. Although there are more than 30 different 96-well sparse matrix screening systems available for soluble proteins, they contain many conditions in which membrane proteins are unlikely to crystallise. The Iwata group have designed crystallisation screens, which are optimised for membrane protein crystallisation. These include two sparse matrix screens based on the available crystallisation conditions at the time of publication (MemStart [26] and MemGold [44^•^]), and a systematic screen (MemSys [26]). Screening the detergent used during crystallisation has proven to be a particularly important aspect of the process [25^•^]. Often crystal quality may also be improved with additional detergents present in the crystallisation drop. Crystallisation of membrane proteins can also be achieved in the presence of lipidic cubic or sponge phases or bicelles instead of detergent micelles [45–47,38^••^]. In the 3D continuous lipidic phases like the cubic and sponge phases, membrane proteins can freely diffuse in the lipid, instead of being enclosed in detergent micelle. The proteins molecules can therefore be concentrated and ordered so that they can form crystals. These techniques have been automated so that even viscous lipidic cubic phase samples can be dispensed in nanodrop quantities [48]. Another technique that is gaining popularity within the membrane protein community is the use of microfluidics to combine very small quantities of protein samples, of the order of 10 nl of protein and crystallisation agent in 50 μm diameter tubes [49,50].

These methods can often lead to crystals that do not diffract beyond 5 Å and are highly anisotropic. Whereas sometimes optimisation of the conditions with the use of additives or other detergents can lead to improved resolution, there remain a large number of crystals for which optimisation is difficult. For these cases it may be necessary to extend the available surface for crystal contacts. This has been achieved in some cases by forming a stable complex with the Fab and Fv fragments of an antibody [51–53,19^••^]. Antibody fragments suitable for co-crystallisation should bind to the protein in its native conformation, have high binding affinity and most importantly bind to a discontinuous epitope. Fab fragments can be produced by proteolytic cleavage of monoclonal antibodies though this sometimes generates heterogeneous products. Since Fabs can also have a high degree of flexibility at the elbow regions between the variable and constant domains [54], Fv fragments may be more suitable for co-crystallisation since they are globular 25 KDa proteins. Another approach to improving the likelihood of crystallisation was taken by Mackinnon and his colleagues in solving the structure of the Kir3.1 K(+) channel. They used chimeras in which part of a eukaryotic protein was replaced by a prokaryotic counterpart [22].

## Data collection and structure solution

Data collection on soluble protein crystals in a high-throughput environment is becoming increasingly routine, with crystals mounted with a sample changer, data collected automatically and structure solution often semi-automated (reviewed in [55^•^]). For membrane protein crystals, the situation is often more challenging. These crystals usually have a high solvent content owing to the detergent micelle, which covers the hydrophobic part of the protein. Consequently, the crystals are often fragile, difficult to handle, diffract to low resolution and suffer from radiation damage during the diffraction experiment. In addition, crystal quality can vary considerably, even between crystals from the same drop. This means that a large number of crystals have to be screened at the synchrotron before data can be collected. The presence of automatic sample changers at most synchrotron beamlines has helped address this issue, enabling many crystals to be screened quickly and efficiently. The use of modern microfocus beamlines with low background scatter and beam sizes of less than 50 μm has also greatly improved the situation [56^•^]. These beamlines can be used to collect datasets from very small crystals and from well diffracting regions in heterogeneous crystals. They can also be used to collect segments of datasets along the length of a crystal, when individual regions suffer from radiation damage [56^•^,43].

The issues mentioned above also affect the process of structure solution. Owing to the problems of radiation damage it may be difficult to collect data of sufficient quality to phase by MAD or SAD. Similarly the problem of non-isomorphism among the crystals can hinder structure solution by the isomorphous replacement method. On the plus side, the high solvent content of membrane proteins can result in solvent flattening giving large improvements in the phases.

## High-throughput methods for membrane proteins

One way to overcome the difficulties associated with membrane protein structural biology is to try a large number of targets and homologues of each target, in the hope that a few will behave relatively well through all these steps. Many soluble protein structural genomics groups have the necessary technology to clone, express and purify many protein targets in parallel, with clones being generated on a 96-well plate scale and crystallisation trials being completed at the rate of 100 crystallisation plates per day [57,58]. Many of these techniques are now being applied to membrane proteins in a number of dedicated membrane protein initiatives.

We have recently established the Membrane Protein Laboratory (MPL) at the Diamond Light Source, UK, which provides a high-throughput environment for researchers from any laboratory in the world to study membrane proteins. The MPL has available a fully automated nanodrop crystallisation system, consisting of a Hamilton Star liquid handler for plate preparation, a Cartesian nanodrop robot for preparation of drops as small as 100 nl, automatic plate sealers and Rhombix imagers at both 4 and 20 °C, which can record 96 images from a crystallisation plate in 10 min. These systems are linked by robotic arms so that up to ten 96-well sitting drop plates can be prepared in one experiment. We also use the Fluidigm Topaz system for microfluidic crystallisation [49] where only limited quantities of protein are available. Potential crystals are screened in crystallisation plates using the PX scanner system, an in-house X-ray system. The MPL is located at the new British synchrotron, Diamond Light Source, which provides a source of intense, highly focused X-rays necessary for crystal screening and data collection on challenging crystals. The Membrane Protein Laboratory is a user facility and anyone can apply to use the equipment and have access the expertise of the membrane protein crystallography (MPC) group at Imperial College London (http://www.diamond.ac.uk/Science/MPL/default.htm).

## Recent advances

One area of membrane protein research in which the methods described here have had a big impact is in the structural biology of G-protein-coupled receptors (GPCRs). GPCRs form the largest family of membrane proteins in humans and their fundamental role in signal transduction has made them very attractive drug targets. They have, however, been notoriously difficult to crystallise, partly owing to their intrinsic flexibility. After many years of work the first structure of a GPCR that is regulated by ligand binding, the b2-adrenergic receptor, was determined past year by two crystallography groups both collaborating with Brian Kobilka [19^••^,38^••^]. Reducing the flexibility of the protein was instrumental in solving the structure of this protein. In one of the structures the third intracellular loop of the protein was stabilized by a Fab fragment [59^••^]. Crystals of the receptor-Fab complex were obtained from bicelles, a mixture of detergents and lipids, and data were collected to a resolution of 3.7 Å at a microfocus beamline from multiple regions of one crystal. The other structure was solved as a fusion protein with T4-lysozyme inserted into the third intracellular loop, again to stabilize this loop [38^••^,60^••^]. The protein was crystallised in a lipidic cubic phase with cholesterol used as an additive. Microbeam technology was used to collect a single dataset merged from 25 microcrystals to a resolution of 2.4 Å. This is reviewed in more detail by Kobilka and Schertler [61].

The structure of the b1-adrenergic receptor has now also been published. In this case, the protein was modified by 6-point mutations chosen on the basis of the increased thermostability of the protein (see above). The effect of these mutations was firstly to render the protein more stable in detergents used for crystallization and secondly to push the conformational equilibrium of the protein towards the antagonist bound state. Data were collected to a resolution of 2.7 Å from crystals of this mutant protein. At the Diamond-MPL, we are also working on structural studies of GPCRs in collaboration with the ERATO human crystallography project (http://cell.mfour.med.kyoto-u.ac.jp/). The microfocus beamline at Diamond will be a powerful tool for solving the structures of membrane proteins including GPCRs.

## Conclusion

It is clear that the structure solution of membrane proteins still holds specific challenges compared with soluble proteins. The field is now, however, taking advantage of the high-throughput revolution in structural biology and is developing a wealth of methods to stabilise and engineer proteins so that they can be crystallised. We expect that in the next five years there will be a rapid growth in the number of solved membrane protein structures, in particular for eukaryotic membrane proteins. This will increase our understanding of the folds and functions in the membrane proteome and provide a wealth of information for the design of novel drugs.

## Conflict of interest

The authors are not aware of any conflict of interest arising from this work.

## References and recommended reading

Papers of particular interest, published within the period of review, have been highlighted as:

• of special interest
•• of outstanding interest
